# Identification of lysyl oxidase as an adipocyte-secreted mediator that promotes a partial mesenchymal-to-epithelial transition in MDA-MB-231 cells

**DOI:** 10.37349/etat.2024.00201

**Published:** 2024-01-16

**Authors:** Cassidy M. Van Stiphout, Grant Kelly, Nikitha K. Pallegar, Eman Elbakry, Ana Valeria Vilchis-Celis, Sherri L. Christian, Alicia M. Viloria-Petit

**Affiliations:** Institute of Experimental Endocrinology and Oncology “G. Salvatore”-National Research Council (IEOS-CNR), Italy; ^1^Department of Biomedical Sciences, Ontario Veterinary College, University of Guelph, Guelph, ON N1G 2W1, Canada; ^2^Department of Biochemistry, Memorial University of Newfoundland, St. John’s, NL A1B 3X9, Canada; ^3^Beatrice Hunter Cancer Research Institute, Halifax, NS B3H 4R2, Canada; ^4^Department of Morphology, National Polytechnic Institute, Mexico City, CDMX 07738, Mexico

**Keywords:** Lysyl oxidase, mesenchymal-to-epithelial transition, metastasis, 3-dimensional culture, extracellular matrix, adipocytes, triple-negative breast cancer, obesity

## Abstract

**Aim:**

Breast cancer (BC) is the most common cancer in women worldwide, where adiposity has been linked to BC morbidity. In general, obese premenopausal women diagnosed with triple-negative BC (TNBC) tend to have larger tumours with more metastases, particularly to the bone marrow, and worse prognosis. Previous work using a 3-dimensional (3D) co-culture system consisting of TNBC cells, adipocytes and the laminin-rich extracellular matrix (ECM) trademarked as Matrigel, demonstrated that adipocytes and adipocyte-derived conditioned media (CM) caused a partial mesenchymal-to-epithelial transition (MET). Given that MET has been associated with secondary tumour formation, this study sought to identify molecular mediators responsible for this phenotypic change.

**Methods:**

Adipocytes were cultured with and without Matrigel, where semi-quantitative proteomics was used to identify proteins whose presence in the CM was induced or enhanced by Matrigel, which were referred to as adipocyte-secreted ECM-induced proteins (AEPs). The AEPs identified were assessed for association with prognosis in published proteomic datasets and prior literature. Of these, 4 were evaluated by the reverse transcription-quantitative polymerase chain reaction (RT-qPCR) and enzyme-linked immunosorbent assay (ELISA), followed by a functional and MET marker analysis of 1 AEP on MDA-MB-231 cells grown on Matrigel or as monolayers.

**Results:**

The 4 AEPs showed a positive correlation between protein expression and poor prognosis. RT-qPCR analysis reported no significant change in AEPs mRNA expression. However, lysyl oxidase (LOX) was increased in CM of ECM-exposed adipocytes. Recombinant LOX (rLOX) caused the mesenchymal MDA-MB-231 TNBC cells to form less branched 3D structures and reduced the expression of vimentin.

**Conclusions:**

The data suggest that adipocyte-secreted LOX changes the mesenchymal phenotype of BC cells in a manner that could promote secondary tumour formation, particularly at sites high in adipocytes such as the bone marrow. Future efforts should focus on determining whether targeting LOX could reduce BC metastasis in obese individuals.

## Introduction

Breast cancer (BC) is the most common cancer in women worldwide, where obesity is one of the major risk factors underlying BC morbidity [1]. Specifically, obese premenopausal women diagnosed with triple-negative BC (TNBC) have the worst prognoses [2]. This subgroup of women tends to have larger tumours with more metastases and respond more poorly to chemotherapy compared to lean women [3–6]. White adipose tissues (WAT) depots occur in visceral and subcutaneous areas as well as within the bone marrow [7–9]. The most common secondary sites of metastasis in BC are bone marrow, lung, liver, and brain, with bone marrow and lung being especially common in TNBC [10]. Obesity increases homing of BC to the bone marrow in the 4T1 mouse model of BC [11]. Moreover, BC cells preferentially interact with adipocyte-rich regions in the bone marrow [12]. This suggests that adipocytes may promote bone marrow metastasis; however, the mechanism is not completely understood [13].

Within the breast and secondary sites of metastasis, the structure and composition of the extracellular matrix (ECM) is altered, providing a suitable environment to support tumorigenesis [14]. In BC, the ECM is highly remodeled where the basement membrane at the primary site is disrupted, directly exposing BC cells that have formed in the duct to other components of the tumour microenvironment (TME) [15]. Furthermore, the nature of the ECM at secondary sites influences the ability of cells to form metastases [16, 17]. Thus, the ECM at both primary and secondary sites can orchestrate crosstalk between BC cells and adipocytes that are in close proximity, resulting in functional and phenotypical changes of both cell types [18]. In exchange, these interactions actively alter the TME to favour tumour initiation, dissemination, and colonization [19].

Cell plasticity is key for tumour progression. During metastasis, epithelial cancer cells have been observed to undergo at least a partial epithelial-to-mesenchymal transition (EMT) to enable migration away from the primary site [20]. Here, epithelial cells progressively lose apical-basal polarity and cell-cell contacts, while acquiring a mesenchymal phenotype and increased migratory and invasive potential [21]. EMT supports cell plasticity, contributing to cell dispersion during development and cancer dissemination [22]. Following this transition, cells may undergo the reverse process, mesenchymal-to-epithelial transition (MET), to allow for growth and colonization at secondary sites, forming metastases [22, 23]. Colonizing cells display MET-like features, including an epithelial phenotype expressing epithelial markers, accompanied by reduced mesenchymal marker expression [24, 25]. Moreover, structural changes occur, as cells revert from elongated forms to adopting more compact and rounded morphology [24].

Evidence shows that cancer cells can successfully metastasize to other organs without completely reverting to an epithelial-like state [26]. The metastasizing tumour cells may exhibit a state of phenotypic duality where tumour cells possess both mesenchymal and epithelial properties. Cells enduring this transitional process are no longer thought to oscillate between the full epithelial and full mesenchymal states, but rather they move through a spectrum of partial/hybrid/intermediate phases [20]. Cells in a partial state have both migratory and cell-cell adhesion properties, promoting the formation of migratory clusters to enhance cell plasticity and survival in different microenvironments [26]. For instance, the multicellular aggregate aids in suppression of senescence and opposition of anoikis, advancing cancer aggression [26, 27]. Thus, a partial tumour state promotes a highly tumorigenic phenotype that ultimately leads to cancer cell stemness, metastasis, and therapeutic resistance [27].

Adipocytes have shown to promote TNBC metastasis by encouraging EMT [28, 29], but have also been implicated in a partial promotion of MET [23, 26]. In a 3-dimensional (3D) culture with laminin-rich Matrigel-based ECM, it was previously demonstrated that mature adipocytes induce a MET-like change in the mesenchymal MDA-MB-231 and Hs578t TNBC cell lines, but they have no effect on SUM159 (mixed morphology) or MCF7 (epithelial) cells [23]. It was additionally found that conditioned media (CM) from mature adipocytes also promotes the MET-like effect, albeit to a reduced efficacy. These findings suggest that adipocyte-secreted factors mediate at least part of this change in morphology that may allow tumour cells to subsequently colonize secondary sites [23]. Comparably, a partial MET was observed in MDA-MB-231 cells cultured with CM from murine WAT [26]. Both lean and obese mouse subcutaneous and visceral WAT-CM caused the TNBC cells to acquire a more epithelial-like morphology [26]. However, the previous study did not examine the influence of bone marrow adipocytes on BC morphology as, to our knowledge, there is no suitable method to isolate and culture mature bone marrow adipocytes from lean and obese mice. Nevertheless, both of the previous studies pointed to the existence of adipocyte-secreted mediators of a partial MET in mesenchymal TNBC [23, 26]. While it has been previously shown that obesity increases the metastasis of BC in a mouse model [11], the exact mechanism(s) by which adipocytes contribute to this colonization are unknown. Next, it was hypothesized that bone marrow adipocytes may induce a partial MET in mesenchymal BC cells to promote colonization. As a first step in identifying the mechanism, the aim of this study was to identify the soluble mediator that promotes the MET-like change in mesenchymal BC cells.

It was first observed that laminin-rich ECM was required for the adipocyte-induced partial MET, so semi-quantitative proteomics was conducted to identify proteins differentially secreted by ECM-exposed adipocytes, which were termed adipocyte-secreted ECM-induced proteins (AEPs). The reverse transcription-quantitative polymerase chain reaction (RT-qPCR) analysis of AEP coding mRNA showed no significant change in mRNA expression of any of the top 4 AEPs [heat shock protein family A member 9 (*HSPA9*), glucosamine-6-phosphate isomerase 1 (*GNPDA1*), V-type proton ATPase subunit E1 (*ATP6V1E1*), and lysyl oxidase (*LOX*)] tested in ECM-conditioned adipocytes. Only the LOX protein was found to be secreted in large quantities by adipocytes, with an enhanced secretion in adipocytes cultured for 48 h with laminin-rich Matrigel-based ECM. Thus, the established 3D culture system was used to assess the effect of human recombinant LOX (rLOX) protein on mesenchymal MDA-MB-231 cells. Imaging showed that the rLOX-treated cells adopted a more epithelial-like morphology, with decreased cellular branching and more compact structures. Further, vimentin expression was also reduced by rLOX treatment. Taken together, the results suggests that LOX, an adipocyte-derived protein, is sufficient to induce an MET-like phenotype in mesenchymal TNBC.

## Materials and methods

### Adipogenesis

To generate mature adipocytes, murine 3T3-L1 pre-adipocytes were differentiated *in vitro*, following the protocol described by the Chemicon^®^ International Adipogenesis Assay Kit instructional manual (cat# ECM950). The 3T3-L1 cell line [cat# CL-173, American Type Cell Collection (ATCC)] was chosen based on our extensive experience with this cell line, and the capacity of differentiated adipocytes to maintain differentiated features in the presence of the mammary epithelial cell growth media used for the 3D co-culture with human BC cells. In addition, the source of ECM used in this co-culture (Matrigel™) is derived from murine cells. 3T3-L1 cells were initially seeded in a 24-well plate at a density of 5 × 10^4^ cells/mL per well and cultured under standard conditions in Dulbecco’s-modified Eagle medium (DMEM; cat# D0819, Sigma Aldrich, Oakville, ON, Canada) supplemented with 10% newborn calf serum (NBCS; cat# N4637, Sigma Aldrich, Oakville, ON, Canada), and 100 IU and 100 μg/mL of penicillin and streptomycin, respectively (cat# SV30010, Thermo Fisher Scientific, Grand Island, NY, USA), which we refer to as DMEM/NBCS.

Adipogenesis was initiated when 3T3-L1 cells reached 100% confluency, by the addition of 0.5 mmol/L 3-isobutyl-1-methylxanthine (IBMX; cat# I7018, Sigma-Aldrich, Oakville, ON, Canada) and 1 μmol/L dexamethasone (cat# D4902, Sigma-Aldrich, Oakville, ON, Canada) in DMEM supplemented with 10% fetal bovine serum (FBS; cat# 12483020, Fisher Scientific, Ottawa, ON, Canada), and 100 IU and 100 μg/mL of penicillin and streptomycin, respectively, which we refer to as DMEM/FBS. Following 48 h incubation, this media was replaced with progression media comprised of DMEM/FBS supplemented with 10 μg/mL insulin (cat# I6634, Sigma-Aldrich, Oakville, ON, Canada). Forty-eight hours later, this media was replaced by DMEM/FBS, where cells were maintained for 5 days, with media changes every other day.

#### Adipocyte-CM and MDA-MB-231 3D culture

To generate CM, mature adipocytes were cultured in 4 different conditions, with media alone (absent of cells) as controls. The conditions varied in the presence or absence of Matrigel-based ECM (growth factor-reduced and phenol red-free Matrigel; cat# CB-40230C, Fisher Scientific Company, Ottawa ON, Canada) as well as media type, either containing DMEM/FBS or supplemented mammary epithelial growth medium (MEGM, cat# C-39110, PromoCell, Heidelberg, DE) (Table 1), which contained 4 μL/mL bovine pituitary extract, 10 ng/mL epidermal growth factor, 5 μg/mL insulin, and 0.5 μg/mL hydrocortisone.

**Table 1:** Culture conditions for the generation of adipocyte CM

**CM conditions**	**Adipocytes**	**Matrigel-based ECM**	**Media type**
1	–	–	DMEM/FBS
2	–	+	DMEM/FBS
3	–	–	Supplemented MEGM
4	–	–	Supplemented MEGM
5	+	–	DMEM/FBS
6	+	+	DMEM/FBS
7	+	–	Supplemented MEGM
8	+	+	Supplemented MEGM

–: exclusion of variable in culture; +: addition of variable in culture

Adipocyte 3D cultures were performed in 24-well plates, with mature adipocytes overlayed with 110 μL of laminin-rich Matrigel-based ECM. For the control conditions, 400 μL of the appropriate culture media was added to the wells. To generate CM from adipocyte cultures unexposed to Matrigel (A-CM), the maintenance media was replaced with 400 μL of the respective CM (see 1–8 in Table 1). For the CM from adipocyte cultures exposed to Matrigel (AE-CM), 392 μL of either DMEM/FBS or supplemented MEGM was mixed with 8 μL of Matrigel (2%) and then served into the wells. After 48 h of incubation, the CM was collected and stored at –80°C for further analysis. Three independent replicates of each condition were included.

To assess potential changes in BC morphology caused by LOX exposure, MDA-MB-231 cells (cat# HTB-26 ATCC, Manassas, VA) were grown as 3D structures on Matrigel, (cat# CB-40230C, Fisher Scientific Company, Ottawa ON, Canada) with added human rLOX protein (cat# CLENZ829-2, Cedarlane Burlington, ON, Canada). A single cell suspension of 1.1 × 10^4^ MDA-MB-231 cells in 450 μL of supplemented MEGM were overlaid on Matrigel. The initial 3D cultures treated with rLOX tested the effect of 0 ng/mL, 25 ng/mL, 50 ng/mL and 100 ng/mL based on a previous study [30], with the greatest morphological change (less branched 3D structures) observed when the cells were cultured with LOX at a concentration of 100 ng/mL for 48 h. This concentration was within the range that would be expected of a 1/2 dilution of AE-CM with fresh media (the protocol we use for treatments with CM), according to the results obtained from the determination of LOX concentration by enzyme-linked immunosorbent assay (ELISA). As such, MDA-MB-231 3D cultures were treated with this concentration for 48 h.

### Immunofluorescence

To generate cultures for immunofluorescence (IF), a solidified layer of Matrigel (100 µL) was added to an empty 4-well chamber slide. Each well was then seeded with MDA-MB-231 cells (30,000 cells/well). To assess the necessity of the ECM in promoting a MET-like change, the control was seeded in supplemented MEGM alone, while the treated were seeded in either AE-CM or A-CM. The treated conditions were diluted 1:2 with supplemented MEGM with 2% Matrigel. After 48 h, the cells were fixed and stained with 4’-6-diamidino-2-phenylindole (DAPI; cat# D1306, Invitrogen, Waltham, MA, USA). The chambers were separated from the slide according to manufacturer’s protocol. An even line of silicone sealant (GE, Boston, MA, USA) was applied around the Matrigel layer to avoid compression of co-cultures by coverslips. ProLong Gold (cat# P10144, Invitrogen, Waltham, MA, USA) was used for mounting slides. The slides were placed in the dark at room temperature to dry overnight. Following, the slides were imaged using the 20× objective with the Nikon A1 confocal microscope with NIS elements imaging software (Nikon Inc, Tokyo, Japan).

IF was also employed to better define the phenotype of LOX-treated MDA-MB-231 cells, in particular the localization of vimentin. For this, MDA-MB-231 cells were cultured as monolayers on 2% Matrigel-coated slides in the absence or presence of rLOX (200 ng/mL) for 48 h, and the cells were then fixed and permeabilized. Following blocking for 1 h at room temperature, the primary antibody for vimentin was applied (1:250; cat# sc-6260, Santa Cruz Biotechnology Inc., Dallas, TX, USA) and cells were incubated overnight at 4°C. This was followed by an incubation with DyLight™ 594 anti-mouse secondary antibody (1:200, cat# A-11005, Thermo Fisher Scientific, Waltham, MA, USA) for 1 h at room temperature. Co-cultures were then mounted with ProLong™ Diamond containing DAPI (Cat# P36971, Thermo Fisher Scientific, Waltham, MA, USA). Absence of the primary or secondary antibody served as negative controls. The slides were imaged at 60× magnification with an oil immersion objective using an Olympus confocal microscope (Model FV500), and processed with the Olympus Fluoview software, version 4.0b (Olympus Canada, Richmon Hill, ON).

### Extracellular vesicle isolation

Extracellular vesicles (EVs) were isolated using ExoQuick (EQPL10TC-1, Systems Biosciences, Palo Alto, CA) following the manufacturer’s protocol. EVs were resuspended in MEGM with 2% Matrigel and applied to MDA-MB-231 cells pre-seeded on Matrigel. EVs were refreshed every 48 h for 5 days. Cells were stained and imaged as above.

### Proteomic analysis

Adipocyte-derived CM was generated as described above under AE-CM and A-CM conditions. The CM was then collected and concentrated through a 10 kDa ultrafiltration unit (Millipore). Protein was separated using a 10% sodium dodecyl-sulfate polyacrylamide gel electrophoresis (SDS-PAGE). Gel followed by silver staining. Gel fragments were sent for mass spectrometry (MS) analysis at the Dalhousie Biological MS Core Facility (Halifax, NS, Canada). Duplicate samples were analyzed using by liquid chromatography-MS (LC-MS)/MS using a VelosPRO Orbitrap (Thermo Fisher Scientific, San Jose, California, USA). Peptide spectra were analyzed using Proteome Discoverer (v2.2, Thermo Fisher). Proteins were considered differentially expressed if more than 2 peptide spectrum matches were assigned and there was a 2-fold or greater increase in AE-CM compared to A-CM for both replicates.

### Survival estimate analysis

Kaplan-Meier (K-M) curves were generated using the K-M plotter online tool, accessible at https://kmplot.com/analysis/. Proteomic data from tumour tissue of BC patients was sourced from two datasets: (HSPA9) Liu et al. [31], and (ATP6V1E1, GNPDA1, LOX) Tang et al. [32]. No restrictions were set on patient subtype, race, menopausal status, or method of treatment. Cut-off for high and low groups was auto-determined as per the creator’s recommendation [33].

### RT-qPCR

Adipocytes were co-cultured with supplemented MEGM in the absence or presence of Matrigel. After 48 h, adipocytes were lysed in TRIzol (cat# 15596026, Ambion, Life Techologies, Carlsbad, CA, USA) and RNA was isolated from TRIzol following the manufacturer’s instructions. RNA was resuspended in RNase free water (cat# 10977-015, Invitrogen, Waltham, MA, USA) and concentrations were determined using the NanoDrop ND-2000 UV-Vis Spectrophotometer (ND-1000, Thermo Fisher Scientific). Samples were then treated with TURBO DNase (Ambion, Life Technologies) using Ambion’s Turbo DNA-free kit (cat# AM1907, Ambion, Life Technologies, Carlsbad, CA, USA) following the manufacturer’s protocol. RNA integrity was verified by gel electrophoresis. Complementary DNA (cDNA) was prepared using M-MLV reverse transcriptase (RT; cat# 28025013, Invitrogen).

Quantitative polymerase chain reaction (qPCR) reactions of 10 µL were carried out using Thermo Fisher Scientific Power SYBRTM Green PCR Master Mix (cat# 4367659, Thermo Fisher Scientific). Five independent replicates were run in duplicate. Reactions were carried out on either an Eppendorf RealPlex 2 system or a Bio-Rad CFX Connect system. Five murine transcripts were examined: 1 normalizer, glucuronidase beta (*GUSB*), and the 4 identified AEPs. All primers were designed and ordered through IDT’s PrimerQuest tool (Table 2). Normalized gene expression was determined using the Pfaffl Method [34]. Statistical significance was determined using a 2-tailed student’s *t*-test. Statistical analysis was completed in GraphPad Prism 8 (GraphPad software, Boston, MA).

**Table 2:** Efficiencies, amplicon length and sequences of qPCR primers used for quantification of murine transcripts

**Gene name**	**Efficiency (%)**	**Amplicon length**	**Forward sequence (5’–3’)**	**Reverse sequence (5’–3’)**	**NCBI accession**
*HSPA9*	92	112 bp	GGGCAAACAAGCAAAGGTCC	TTGCCGTTTTGCTGGCATAC	NM_010481
*GNPDA1*	142	170 bp	GGACGCCACCTTGGAACTAA	GAATACCACGTCCTGGCACA	NM_011937
*ATP6V1E1*	102	87 bp	ATAGCCCAGCAGATGATGCC	TCCTCCTGAAGCTCAGTCCA	NM_007510
*LOX*	103	165 bp	CAGGAACCGACCTGGATACG	GCCCTATATGCTGAACTGGC	NM_010728
*GUSB*	91	108 bp	CAGCGGCTGGGCTTTTTA	CGCTTGCCCTCAACCAAGTT	NM_010368

NCBI: National Center for Biotechnology Information

### LOX ELISA and immunoblotting

The murine LOX ELISA (MBS2512845, MyBiosource, San Diego, CA) was conducted according to the manufacturer’s instructions. The relative absorbance was read at 450 nm using the Agilent BioTrek Synergy HTX Multi-Mode Microplate Reader (Thermo Fisher Scientific, Nepean, Canada).

To evaluate the impact of LOX on MET, 5 × 10^5^ cells were seeded in 6-well plates. The wells were incubated with DMEM + 2% Matrigel at room temperature for 1 h (to permit ECM attachment to the wells). After a single wash with DMEM, MDA-MB-231 cells resuspended in supplemented MEGM were added to the wells, either remaining untreated (negative control) or treated with rLOX (200 ng/mL) for 48 h. In separate wells, NMuMG cells in DMEM/FBS + 10 μg/mL insulin were directly seeded as positive controls, either remaining untreated or treated with transforming growth factor-β (TGF-β, 10 ng/mL) for 48 h, to induce EMT. To lyse, dishes were placed on ice and washed once with phosphate buffered saline (cat# 311-010-CL,Wisent Inc, Montreal, QC, Canada), after which a commercially available lysis buffer (cat# 9803S, Cell Signaling Technology, Danvers, MA, USA) containing 2 μg/mL aprotinin (cat# A6106 Sigma Aldrich, St. Louis, MO, USA), 1% phosphatase inhibitor cocktail (cat# P5726 Sigma Aldrich, St. Louis, MO, USA), 1 mmol/L sodium orthovanadate (cat# P0758L, New England Biolabs, Ipswich, MA, USA) and 1 mmol/L phenylmethylsulfonyl fluoride (cat# P7626, Sigma Aldrich, St. Louis, MO, USA), was added to the dishes. The collected lysates were incubated on ice for 30 min, vortexing every 10 min, then centrifuged at 15,000 *g* for 20 min at 4°C. The supernatant was collected, and protein concentration was determined with a detergent compatible (DC) protein assay using known concentrations of bovine serum albumin for the construction of a standard curve, as per manufacturer instructions (cat# 5000002, BioRad, Hercules, CA, USA).

Twenty micrograms of protein were used for the detection of E-cadherin, zonula occludens-1 (ZO-1), vimentin, and β-actin. Lysates were resolved on 7.5% polyacrylamide. The protein was then transferred to an activated 0.45 μm polyvinylidene fluoride membrane (cat# IPVH85R, Sigma-Aldrich, Oakville, ON, Canada). Membranes were all blocked with 5% non-fat skim milk (M7409, Sigma-Aldrich, Oakville, ON, Canada) powder diluted in tris-buffered saline (TBS; T6664, Sigma-Aldrich, Oakville, ON, Canada) containing 0.1% Tween-20 (TBST; T9039, Sigma-Aldrich, Oakville, ON, Canada) for 1 h at room temperature. Membranes were incubated with the respective primary antibody overnight at 4°C with rocking. The following day, membranes were washed 3 times with TBST and incubated with respective secondary antibodies diluted in 5% milk TBST for 1 h rocking at room temperature. Membranes were washed 3 times with TBST, and Immobilon® Forte Western HRP Substrate (cat# WBLUF0100, Millipore Sigma, Burlington, MA, USA) was applied and membranes were imaged on a ChemiDoc MP Multiplex system withwith ImageLab software (BioRad, Hercules, CA, USA).

Primary antibody concentrations used were as follows: E-cadherin (1:1,000; cat# sc-8426), ZO-1 (1:1,000; cat# sc-33725), and vimentin (1:1,000; cat# sc-6260) were all from Santa Cruz Biotechnology Inc. (Dallas, TX, USA) and were used at a final concentration 0.2 µg/mL. The β-actin (1:5,000; cat# 4967) used as a loading control was from Cell Signalling Technologies (Whitby, ON, Canada). Secondary antibodies used were from Sigma-Aldrich (St. Louis, MO, USA) and were horseradish peroxidase (HRP) conjugated goat anti-rat (1:10,000; cat# A-9037), anti-mouse (1:10,000; cat# A-0168), and anti-rabbit (1:10,000; cat# A-0545).

### Statistical analysis

Morphological changes were assessed using ImageJ v. 1.48 (NIH Image, Bethesda, MD; https://imagej.nih.gov/ij/download.html). A 3D image was pre-processed to extract the relevant morphological information by binarizing the image, removing any potential noise. Briefly, a threshold was set that divided the image into 2 classes of pixels, the foreground and background, outputting the cellular structures in the foreground. Any structures that were not identified during this process were then selected into the foreground manually. To assess colony morphologies, a total of 3 independent experiments with 5 fields of view each were analyzed. A circularity measurement classified the structures into the following groups: round/mass-like (> 0.7), grape-like (0.2−0.7), and stellate (< 0.2). Mean percentage of area covered by each structure type was determined for each condition. For the branching analyses, branches present in 4 images per replicate were traced by using the Skeletonize3D plugin. The number of branches were extracted by skeletonizing the binary image with Skeletonize3D. Analyses of the resulting skeleton in the 3D image was completed with the AnalyzeSkeleton plugin.

Statistical differences between conditions were determined using Prism. An unpaired *t*-test was used for all analyses.

## Results

### MET-mediating factor is an AEP

To identify the adipocyte-derived mediator causing the MET-like effect, the influence of secretory factors on MDA-MB-231 cell morphology was investigated. It was found that neither small molecules (< 10 kDa) nor EVs were able to promote an epithelial like morphology in these cells (Figure S1). Therefore, it was concluded that it is most likely free protein(s) that is/are mediating this effect.

**Figure 1:**
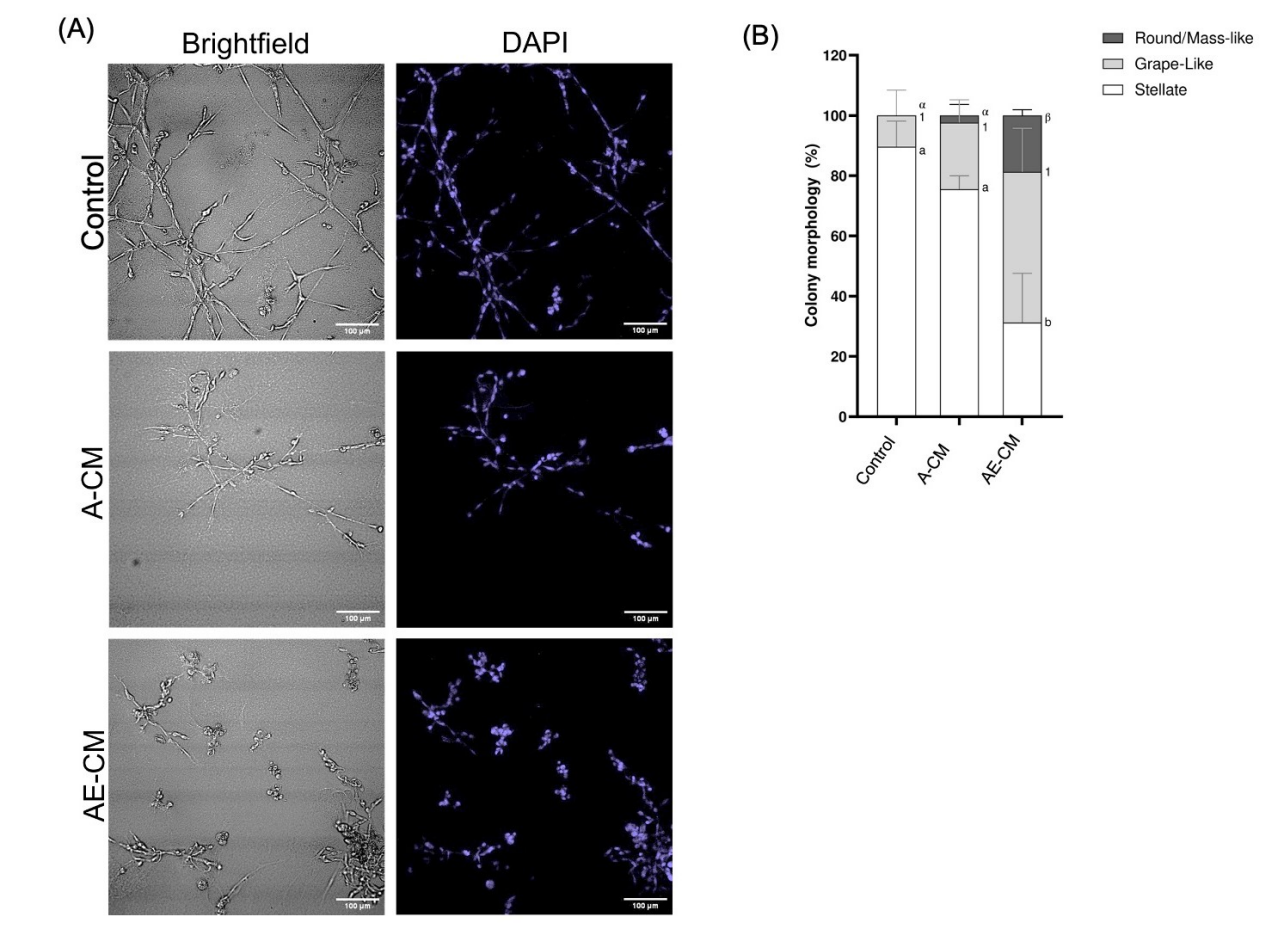
ECM is required for adipocytes to promote a partial MET in MDA-MB-231 cells grown in 3D. (A) Representative brightfield and DAPI images of MDA-MB-231 cells grown in 3D with either adipocyte-CM treated with ECM (AE-CM) or without ECM (A-CM). For both conditions, adipocytes were grown in supplemented MEGM for 48 h prior to CM collection. Images taken at 200× total magnification; (B) total percentage of cell structure shapes, reported as mean ± standard error of the mean (SEM). Significance of differences between groups was determined by one-way ANOVA. Data was derived from 3 independent replicates. Letters, numbers and symbols represent statistically different groups: a, b: stellate; 1: grape-like; α, β: round/mass-like

Next, it was addressed whether the ECM was a necessary factor in promoting the epithelial morphology. To do this, 3T3-L1 preadipocytes were fully differentiated to mature adipocytes. Differentiation was confirmed when at least 40% of the adipocytes in culture had obtained lipid droplets (Figure S2). Mature adipocytes were then cultured in supplemented MEGM with (AE-CM) or without (A-CM) the ECM layer, followed by collection of the CM at 48 h. It was observed that the MDA-MB-231 cells cultured with AE-CM displayed the expected gain of round/mass-like structures and loss of stellate structures, while the A-CM showed no significant difference relative to the control (*P* < 0.05) (Figure 1). Therefore, this indicated that the Matrigel-based ECM was required for adipocytes to promote a partial MET. Subsequently the protein(s) causing this MET-like effect, were referred to as, AEPs.

**Figure 2:**
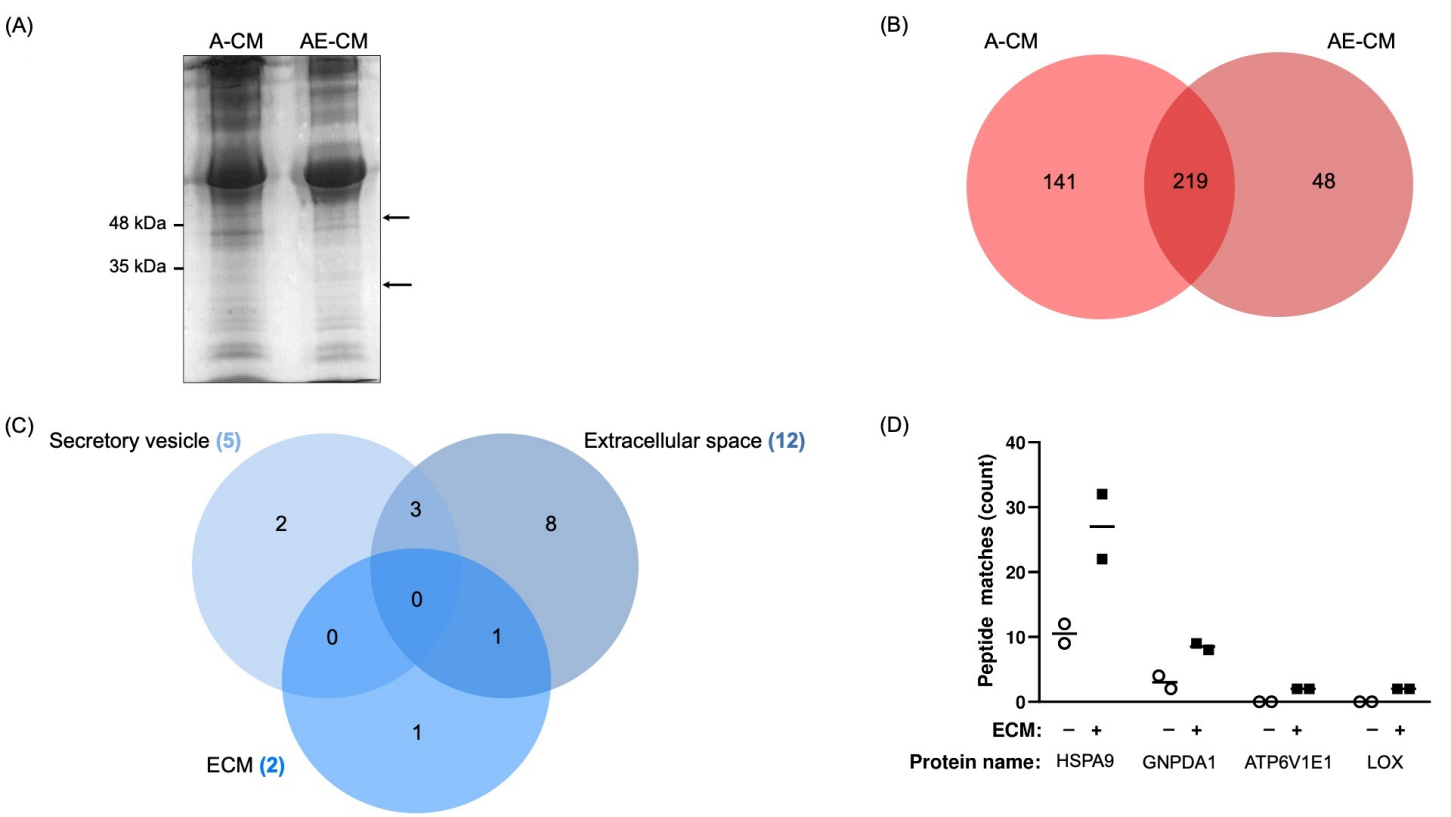
Identification of proteins with increased expression in adipocyte CM in the presence of ECM (AE-CM) revealed a top 4 list. (A) SDS-PAGE of adipocyte CM (A-CM) and AE-CM reveals two protein bands exclusive to AE-CM. Molecular weight markers are shown to the left of the gel and the two differentially expressed bands are indicated with arrows. Data are representative of two independent replicates; (B) in a semi-quantitative proteomic analysis, 141 unique proteins were found in A-CM, 48 distinctive proteins were exclusively found in AE-CM, and 219 proteins were found in both conditions; (C) cellular localization of differentially expressed proteins. Note that only 19 are shown as we specifically examined secretory vesicle, extracellular space and ECM; (D) relative abundance of 4 differentially secreted proteins by number of peptide spectrum matches. Two independent replicates were analyzed. +/–: cultures without (–) or with (+) the Matrigel-based ECM

Next, we used proteomics to identify proteins that were only found in the AE-CM. The A-CM or AE-CM was separated by gel electrophoresis and two bands were found to be present in the AE-CM that were not present in the A-CM (Figure 2A). We then performed semi-quantitative proteomics and found 102 proteins with increased levels in AE-CM. Of these, 48 proteins were unique to AE-CM (Figure 2B). We then compared the list of proteins either exclusive to AE-CM or increased by at least 2-fold [with at least 2 peptide spectrum matches (PSM)] to the composition of Matrigel reported by Hughes et al. [35]. This reduced the list to 33 unique proteins (Table S1). Analysis of the cellular localization of these proteins revealed that only one protein is found in the extracellular space, not in secretory vesicles, and is associated with ECM (Figure 2C). This protein is LOX. In the end, the possibilities were narrowed to 4 proteins based on their known secretion profile and literature review (Figure 2D).

### AEPs are associated with worse prognostic outcomes in BC patients

**Figure 3:**
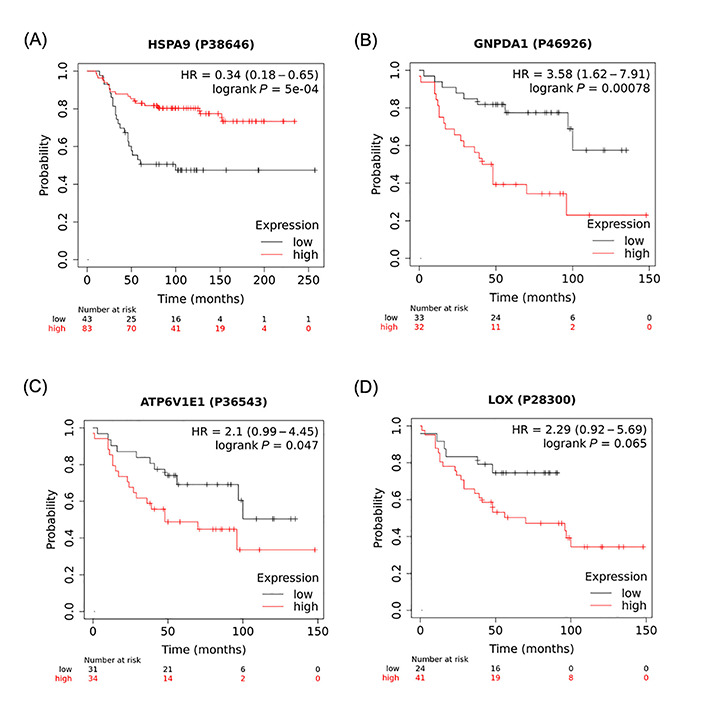
K-M plots depicting a positive association between high expression of identified proteins and overall survival of BC patients. A strong correlation was found between the number of months to live in BC patients that expressed high levels of (A) HSPA9; (B) GNPDA1; (C) ATP6V1E1; and (D) LOX when compared to patients that expressed low levels of these proteins. These data were analyzed using https://kmplot.com/analysis/ with the original datasets sourced from (B, C, D) Tang et al. [32] and (A) Liu et al. [31] and have not been previously published in this form. P38646, P46926, P36543, P28300 are GeneIDs (from UniProtID); HR: hazard ratio

Analysis of the K-M plots of these 4 proteins revealed that high levels of all four proteins, HSPA9, GNPDA1, ATP6V1E1, and LOX, in tumour tissue of BC patients, are associated with worse survival when all BC types were considered (Figure 3). This association was statistically significant for HSPA9, GNPDA1, ATP6V1E1, and borderline significant for LOX.

### LOX secretion increases in CM from ECM exposed-adipocytes

**Figure 4:**
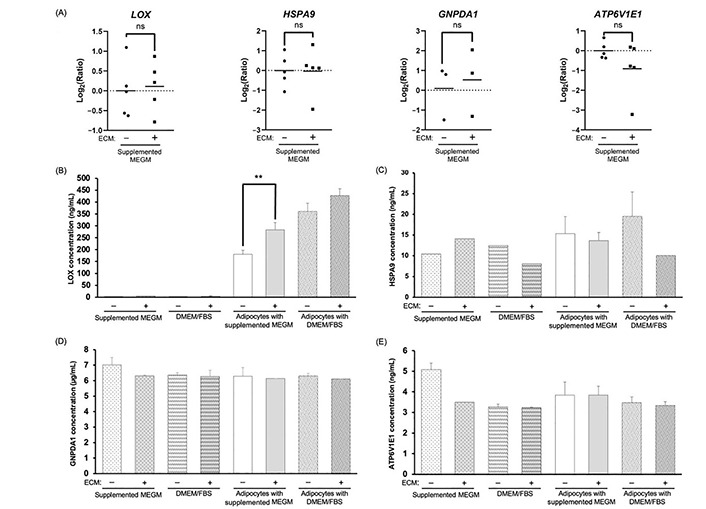
Evaluation of mRNA and protein for the four AEPs shows enhanced LOX protein in adipocyte-derived CM. (A) RT-qPCR analysis of the top 4 AEPs mRNA expression; (B–E) representative bar graphs depicting the average concentration of the top 4 AEPs in the different CM; average concentration values for (B) LOX are from 3 independent replicates; significance of differences between adipocyte-derived conditions was determined by an unpaired, two-tailed *t*-test, which was applied to each type of media; (C) HSPA9; (D) GNPDA1; and (E) ATP6V1E1 are from 2 independent replicates, except for the 5 conditions with missing error bars for HSPA9, where *n* = 1. +/–: cultures without (–) or with (+) Matrigel-based ECM; ns: non-significant. ^**^ *P* < 0.01

We next quantified the mRNA expression of the 4 AEPs via RT-qPCR, comparing AE-CM and A-CM conditions. Surprisingly, none of the AEPs showed a significant change in mRNA expression between conditions (Figure 4A).

Next, we measured AEP secretion at 48 h. Of the 4 AEPs, changes in protein secretion between conditions was only observed for the LOX protein, where LOX levels increased in CM compared to media alone controls (Figure 4B–E). At 48 h, the average LOX concentration was approximately 312.9 ng/mL in adipocyte-derived CM compared to approximately 3.4 ng/mL in cell-free CM. The CM of adipocytes cultured in the presence of the Matrigel-based ECM contained higher levels of LOX compared to CM of ECM-free adipocytes, and this was only significant for CM of adipocytes cultured in MEGM (Figure 4B; *P* = 0.007).

### LOX reduces invasive branching of TNBC cells grown in 3D culture

Next, we tested the effect of human rLOX on TNBC cells grown as 3D structures to determine if LOX is sufficient to modify TNBC morphology.

**Figure 5:**
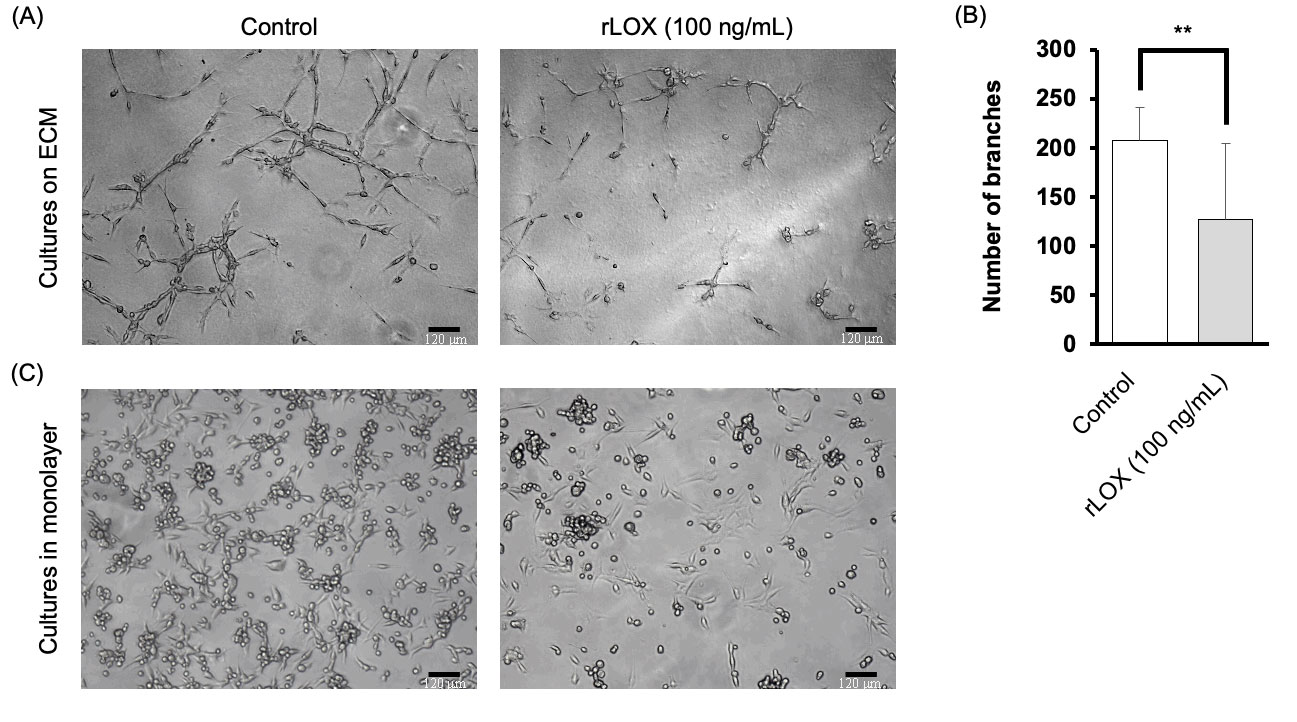
rLOX decreases invasive branching of multicellular MDA-MB-231 structures. (A) Representative images of MDA-MB-231 3D cultures on Matrigel. Single cell suspensions were plated in the absence or presence of 100 ng/mL of rLOX and cultured for 48 h. Magnification = 100×, scale bar = 120 μm; (B) average number of branches of MDA-MB-231 stellate structures on Matrigel significantly decreased in the presence of rLOX by approximately 80 branches (*P =* 0.008). Significance of differences between conditions was determined by an unpaired, two-tailed *t*-test. Averages were obtained from 3 independent replicates, with 2 technical replicates per condition. The number of branches were calculated based on 4 images per technical replicate; (C) representative images of MDA-MB-231 cell monolayer cultures on glass in the absence or presence of rLOX (100 ng/mL). ^**^ *P* < 0.01

Based on the literature, we tested varying concentrations of rLOX: 25 ng/mL, 50 ng/mL and 100 ng/mL diluted in supplemented MEGM [30]. After 48 h, it was observed that MDA-MB-231 multicellular structures exhibited morphological changes when cultured with rLOX at 100 ng/mL (Figure 5A). In the presence of rLOX, the TNBC cells formed densely populated clusters relative to other regions that showed little to no cell growth. Quantitative analysis of stellate structures formed on Matrigel corroborated our observations, where the presence of rLOX promoted a significant decrease in invasive branching of BC cells (Figure 5B).

This change in morphology provided incentive to further investigate MET markers. As such, cells were then cultured in monolayer, using supplemented MEGM as the culture media, to verify that at least some of the phenotype could be reproducible in 2D. The rLOX-treated monolayers showed a reduced cell density, with less of the surface area occupied by cells, as well as less foci of rounded cells (Figure 5C).

### LOX has a partial effect on expression of MET markers in MDA-MB-231 cells

We then further characterized the MET-like effect by immunoblotting to specifically detect the expression of MET markers in a semi-quantitative manner. We assessed the expression of E-cadherin and ZO-1, both epithelial markers, and vimentin, a mesenchymal marker in cell lines NMuMG and MDA-MB-231 cell lines. The NMuMG cells were treated without and with TGF-β, both conditions acting as a positive control for epithelial marker expression. TGF-β is known to induce EMT, thus demonstrating a decrease in epithelial marker expression. In addition, MDA-MB-231 cell monolayers grown on Matrigel-coated plates were cultured in the absence or presence of rLOX (200 ng/mL). This protein concentration is still within the range of LOX concentration detected by the ELISA in the CM of adipocytes cultured with overlaid Matrigel in supplemented MEGM, and it was chosen to account for the reported enhanced activation of signalling mediators potentially modulated by LOX in 2D *versus* 3D cultures [36].

**Figure 6:**
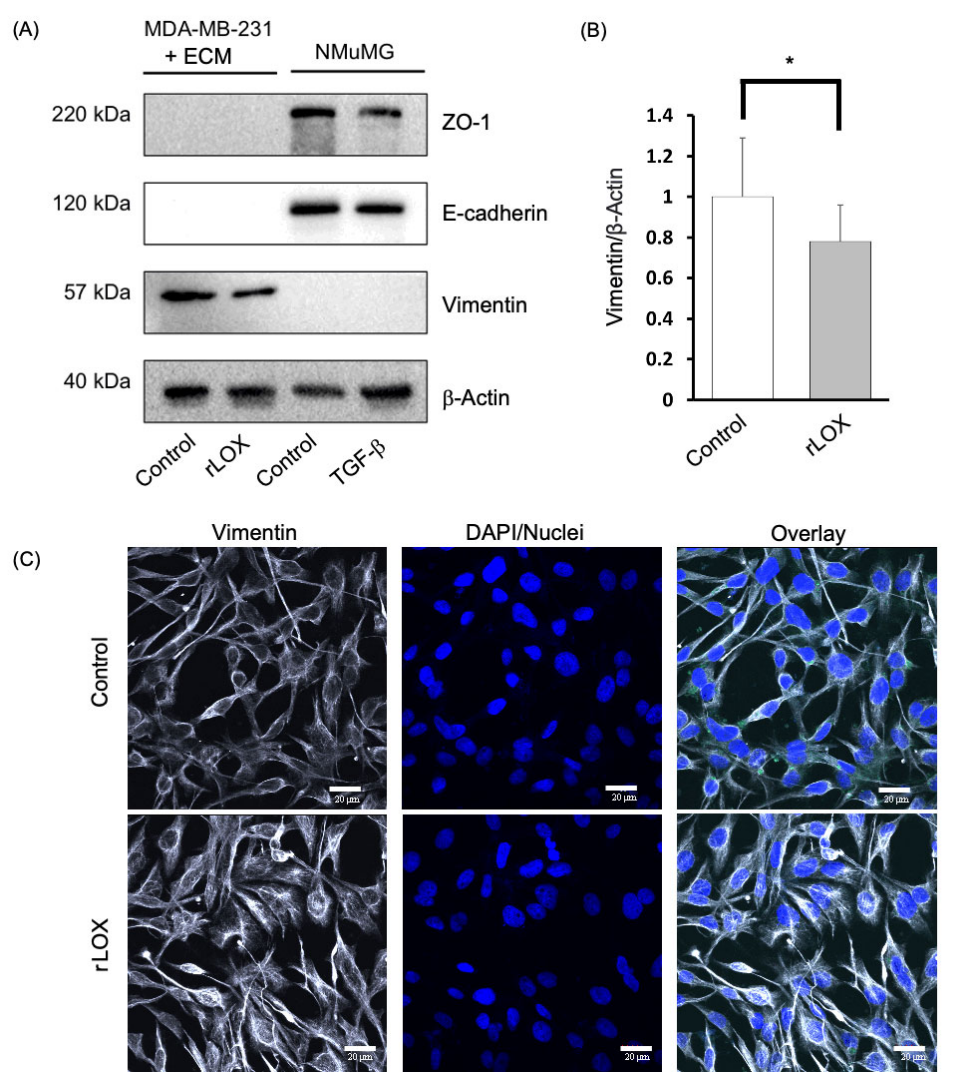
rLOX reduces the expression of vimentin in MDA-MB-231 cells. (A) MET markers and β-actin (loading control) were assayed via immunoblotting in TNBC MDA-MB-231 cells and immortalized NMuMG cells, which were used as controls for epithelial markers using antibodies that recognize both, the human and mouse proteins. The MDA-MB-231 and NMuMG cells were treated with rLOX (200 ng/mL) and TGF-β (10 ng/mL), respectively for 24 h; (B) relative vimentin protein expression in MDA-MB-231 cells significantly decreased by more than 20% in the presence of rLOX (200 ng/mL) compared to the control (*P* = 0.0382). Significance was determined using an unpaired, one-tailed *t*-test with Welch’s correction. (A) and (B) data was derived from 3 independent replicates. ^*^P < 0.05; (C) representative images of MDA-MB-231 cells growing as monolayers on 2% Matrigel in the absence of presence of rLOX and co-stained with anti-vimentin and DAPI. IF observations were derived from 1 experiment. Magnification = 600×, scale bar = 20 μm

As expected, NMuMG cells only expressed the epithelial markers E-cadherin and ZO-1, whereas untreated MDA-MB-231 cells only expressed the mesenchymal marker, vimentin. Interestingly, we observed that the rLOX treatment reduced vimentin expression but did not induce the expression of E-cadherin nor ZO-1 (Figure 6A). Densitometry analysis revealed that vimentin expression decreased by more than 20% compared to the MDA-MB-231 control (Figure 6B). To localize the MET markers and better define the phenotype, we subsequently performed IF. Similar to the control, the rLOX treated cells showed no epithelial marker expression (data not shown). Vimentin was detected in both conditions, with rLOX treatment showing a more dispersed cytoskeleton compared to the control (Figure 6C). Together, these data suggest that LOX alters the levels and localization of vimentin in MDA-MB-231 cells. LOX may be pushing MDA-MB-231 cells towards a partial MET-like state, where there is neither the complete loss of mesenchymal markers nor the complete gain of epithelial markers.

## Discussion

In this study, we sought to identify the factor secreted by adipocytes that is responsible for inducing a partial MET in BC cells. First, semi-quantitative proteomics identified proteins whose presence in adipocyte-derived CM was induced or enhanced by Matrigel-based ECM. These proteins, which we referred to as AEPs, were further evaluated to assess their association with overall survival in BC patients. Based on secretion profiles, high levels of 4 AEPs were correlated with a worsened prognosis in BC patients, namely HSPA9, GNPDA1, ATP6V1E1, and LOX. LOX was the only one of the AEPs that did not reach statistical significance (*P* = 0.065). This could be because the source data included all BC subtypes. Nevertheless, in support of a role for LOX in metastasis, Saatci et al. [37] found a statistically significant difference (*P* = 0.019) in disease-free survival (DFS) over 150 months in 77 TNBC patients that received chemotherapy, where the patients with higher LOX levels have the shortest DFS. These findings prompted us to narrow our investigation, focusing on these 4 AEPs to determine if any were involved in promoting the observed MET-like effect.

Interestingly, LOX was the only AEP present in substantially larger quantities in the CM of adipocytes, with increased expression in the presence of Matrigel (Figure 4). LOX is a member of the LOX family, a type of secreted copper-dependent amine oxidases, which is comprised of LOX and four LOX-like (LOXL1**–**4) isoenzymes [38]. There is 99% identity between human and mouse LOX at the protein level. LOX/LOXL are defined as key enzymes that regulate ECM homeostasis [38]. LOX, the main isoenzyme and best characterized, plays well-documented roles in the crosslinking of elastin and collagen fibres, the modulation of ECM structure, as well as the regulation of cell differentiation and migration [39–42]. The upregulation of LOX activity, for example, has been linked to fibrosis; a pathological feature associated with adipose tissue dysfunction, where a surplus of insoluble collagens in the ECM increases tissue stiffness and impairs organ function [39]. Moreover, in 3T3-L1 cells, LOX-propeptide (LOX-PP) has also shown to prevent the inhibition of adipogenesis by fibroblast growth factor-2 [42]. Interestingly, LOX has been reported as the main isoenzyme expressed in human adipose tissue, and its expression in adipose tissue is significantly higher in obese patients and obese rats [40]. As such, LOX may play a critical role in fostering a metabolically dysfunctional microenvironment.

Our results indicate that Matrigel may facilitate the secretion of LOX. Both media types, DMEM/FBS and supplemented MEGM, showed an increase in LOX production in the presence of Matrigel. Cultures with Matrigel have shown to advance 3T3-L1 cell growth and differentiation [43]. Interestingly, Matrigel-exposed adipocytes in this study had an evident reduction in lipid droplet size (Figure S2). This was also observed in the previous study by our group [23], and could represent changes in lipid metabolism (enhanced catabolism or reduced anabolism) induced by ECM-derived signaling. The influence of ECM on lipid metabolism has been previously reported [44, 45].

In 3D culture, we found that human rLOX protein reduced the invasive branching of MDA-MB-231 cells. Invasive branching is mediated by mechanical forces, wherein the tip of a new epithelial branch induces tensile forces on the surrounding tissue to protrude forward [46]. At the same time, the cells that lag behind the tip cell, sometimes referred to as “follower cells”, “trailing cells” or “stalk cells”, maintain adhesion to each other as well as to the tip cell, generating sufficient pushing forces to move the collective forward [46]. Indeed, a primary regulator of branching morphogenesis is the microenvironment [47, 48]. Numerous biochemical signals, including growth factors, proteases, and ECM molecules act as global regulators of branching morphogenesis in culture and *in vivo* [47]. In cancer, where the TME is highly remodeled, changing mechanical properties such as the crosslinking of the surrounding ECM, can regulate branching formation [48]. LOX activity, for example, can increase the stiffness of the ECM [48]. These external changes in mechanical forces can then result in the compression or confinement of the tumour itself, reducing cancer cell proliferation while also enhancing their metastatic potential [49].

LOX has been found to not only modulate the behaviour of local cells at the primary site, but also at distant secondary sites prior to tumour cell arrival [37, 50]. For instance, LOX secretions from cancer cells are capable of remodeling local environments, where it creates permissive niches (premetastatic niches) for tumour cells to subsequently colonize and form overt secondary tumours [41, 51, 52]. Joo et al. [53] found that nude mice injected with MDA-MB-231 cells showed high levels of LOX secretion, followed by enhanced crosslinked collagen and CD11b^+^ bone marrow-derived cell recruitment, ultimately forming a receptive microenvironment for colonization in the lungs. Our data demonstrates the tendency of rLOX treated MDA-MB-231 cells to localize and form densely populated clusters, resembling structures of newly colonized, secondary tumour sites. As such, the secretion of LOX not only by BC cells, but also potentially by stromal cells like adipocytes, could play a critical role in premetastatic niche formation and the likelihood of disseminated cancer cells to colonize secondary tumour sites.

Colonizing cells tend to regain epithelial-like morphologies, organizing into tightly connected multicellular layers with strong cell-cell junctions on both lateral sides that maintains apical-basal polarity [54, 55]. Moreover, cell-matrix adhesions anchor the cells to the basement membrane, therefore limiting cell motility [55]. It is possible that LOX-induced invasion and metastases is mediated, in part, by its effects on the structure and physical properties of the ECM. Under normal conditions, an optimal degree of LOX-dependent cross-linking maintains the basement membrane and the stromal compartment of the ECM [56]. However, under pathologic conditions such as obesity, LOX is upregulated leading to dysregulation in matrix remodeling that generates a positive pro-obesity feedback loop [40]. It is possible that this feedback loop plays an important role linking obesity to BC progression.

The classic description of MET is characterized by the following: a complete acquisition of epithelial morphology, when mesenchymal proteins are downregulated, and epithelial proteins are upregulated [21]. In this study, we found that the rLOX treatment moderately downregulated the expression of vimentin, a mesenchymal biomarker, without inducing the expression of epithelial markers in MDA-MB-231 cells. As such, it appears that the MDA-MB-231 cells are in a partial MET state. Gunasinghe et al [54] have made similar observations using an MDA-MB-468 xenograft model where BC cells undergoing local lympho-vascular invasion transitioned from a mesenchymal/epithelial state to an epithelial state. They found that the tumour emboli entered an intermediary state in which cells gradually lost vimentin expression and shifted towards a phenotype that predominantly expressed E-cadherin [54]. This evidence supports the idea that the intermediary phenotype observed in our work could be a transitionary phase, potentially followed by further downregulation of vimentin and possibly other mesenchymal markers, as well as amplification of epithelial markers.

Here, we demonstrated that rLOX is sufficient to drive a MET-like change in MDA-MB-231 cells. This expands on our prior work [23, 26], confirming that a specific adipocyte-derived mediator, in this case LOX, modulates the TNBC phenotype in an ECM-dependent manner that could be responsible, at least in part, for LOX role in BC metastasis. In agreement with this possibility, bone marrow-derived mesenchymal stem cells have been shown to stimulate de novo LOX production in MDA-MB-231 and MCF7 BC cell lines, which correlated with enhanced lung and bone metastases in a mouse model [57]. Future efforts could be directed towards inhibiting LOX activity. More recently, the use of LOX inhibitors, such as β-aminopropionitrile (BAPN) and derived compounds, have been recognized to limit metastases while also controlling body weight [40, 58]. Miana et al reported that BAPN reduces body weight gain and improves the metabolic profile in rats with diet-induced obesity when orally administered (100 mg/kg bw/d) [40]. In this study, this inhibitor also attenuated the increase in WAT fibrosis [40]. Moreover, Saatci et al. [37] found that inhibiting LOX in TNBC tumours *in vivo* reduced collagen crosslinking and increased drug penetration, resulting in the induction of apoptosis and re-sensitization to chemotherapy.

In summary, we identified LOX as a protein secreted by adipocytes that induces a partial MET-like effect in the MDA-MB-231 TNBC cell line. Our work suggests that targeting LOX could serve as a promising anti-cancer treatment in obese patients. However, more work is required to demonstrate the relationship between obesity, LOX and BC metastasis.

## Abbreviations

3D: 3-dimensional
A-CM: conditioned media from adipocytes unexposed to Matrigel
AE-CM: conditioned media from adipocytes exposed to Matrigel
AEPs: adipocyte-secreted extracellular matrix-induced proteins
ATP6V1E1: V-type proton ATPase subunit E1
BC: breast cancer
CM: conditioned media
DAPI: 4’-6-diamidino-2-phenylindole
DMEM: Dulbecco’s-modified Eagle medium
ECM: extracellular matrix
ELISA: enzyme-linked immunosorbent assay
EMT: epithelial-to-mesenchymal transition
EVs: extracellular vesicles
FBS: fetal bovine serum
GNPDA1: glucosamine-6-phosphate isomerase 1
HSPA9: heat shock protein family A member 9
IF: immunofluorescence
K-M: Kaplan-Meier
LOX: lysyl oxidase
MEGM: mammary epithelial growth medium
MET: mesenchymal-to-epithelial transition
MS: mass spectrometry
rLOX: recombinant lysyl oxidase
RT-qPCR: reverse transcription-quantitative polymerase chain reaction
TBST: tris-buffered saline containing 0.1% Tween-20
TGF-β: transforming growth factor-β
TME: tumour microenvironment
TNBC: triple-negative breast cancer
WAT: white adipose tissue
ZO-1: zonula occludens-1

## Supplementary materials

The supplementary material for this article is available at: https://www.explorationpub.com/uploads/Article/file/1002201_sup_1.pdf.

## Declarations

### Availability of data and materials

The data are available from the authors upon request (sherri.christian@mun.ca; aviloria@uoguelph.ca).

### Copyright

© The Author(s) 2024.
