# Supplementary material for: Identification of lysyl oxidase as an adipocyte-secreted mediator that promotes a partial mesenchymal-to-epithelial transition in MDA-MB-231 cells
Source: Explor Target Antitumor Ther. 2024 Jan 16;5(1):1–19. doi: 10.37349/etat.2024.00201 (PMC10927314; doi:10.37349/etat.2024.00201)
Supplement: Supplementary file 1 [file 1002201_sup_1.pdf]

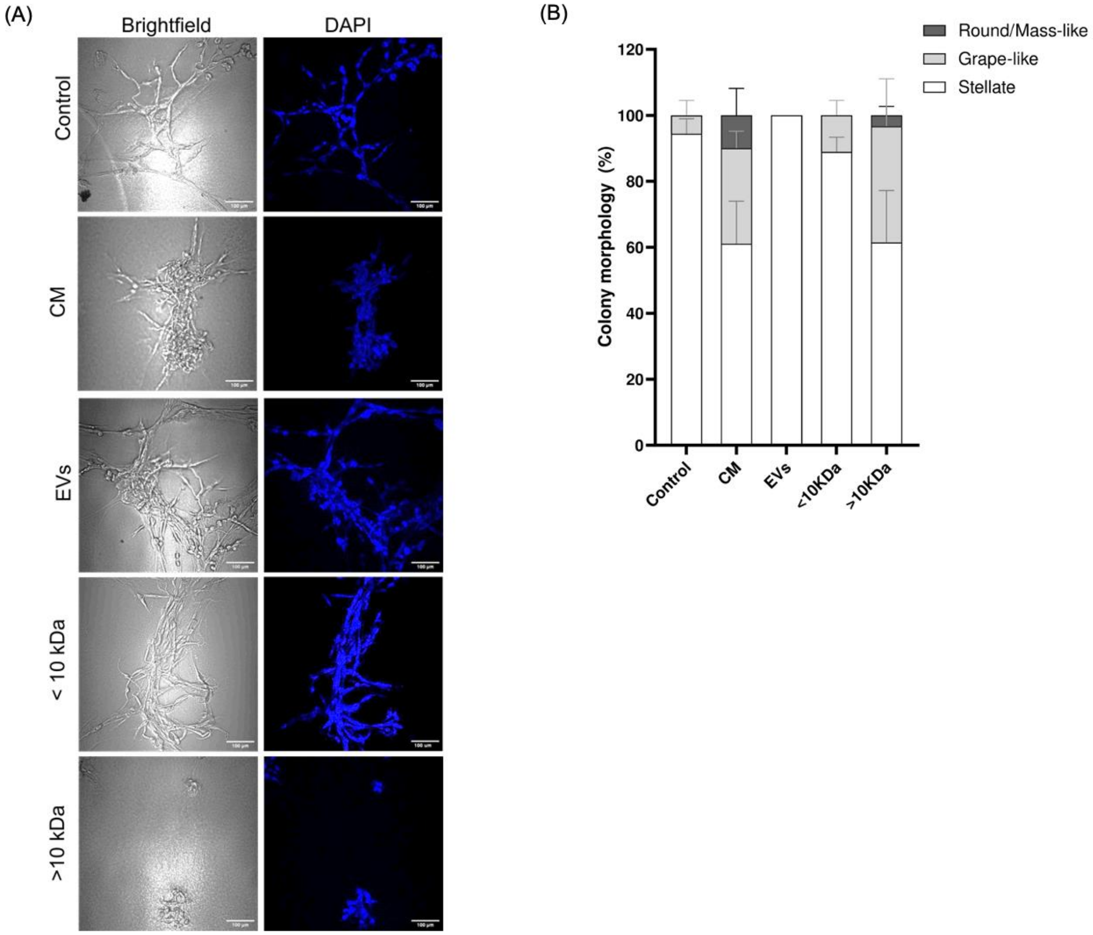

Figure S1. Identification of free proteins as the adipocyte-derived mediator causing the MET-like effect in MDA-MB-231 cells. (A) Representative images of MDA-MB-231 cells grown in Matrigel and treated with AE-CM containing particles greater or less than 10 kDa in size or containing only EVs. EVs were precipitated from AE-CM using the ExoQuick EV isolation kit (System Biosciences, Palo Alto, CA). To separate AE-CM into <10 kDa or >10 kDa fractions, collected AE-CM was subjected to ultracentrifugation at 14,000 *g* for 30 minutes in a 10 kDa ultrafiltration spin column. This was then repeated at 10,000 *g* for 1 minute, using a fresh collection tube. 500 µL of the < 10 kDa and > 10 kDa fractions were collected from the elute and concentrate remaining in the column, respectively. These were diluted 1:2 in growth media before addition to MDA-MB-231 cultures; (B) total percentage of cell culture structures, reported as mean ± SEM. Differences between the groups approached statistical significance however, due to insufficient experimental repetitions, significance could not be reached. Structures were determined by visual identification in ImageJ. Graph and Images prepared using GraphPad Prism and ImageJ respectively

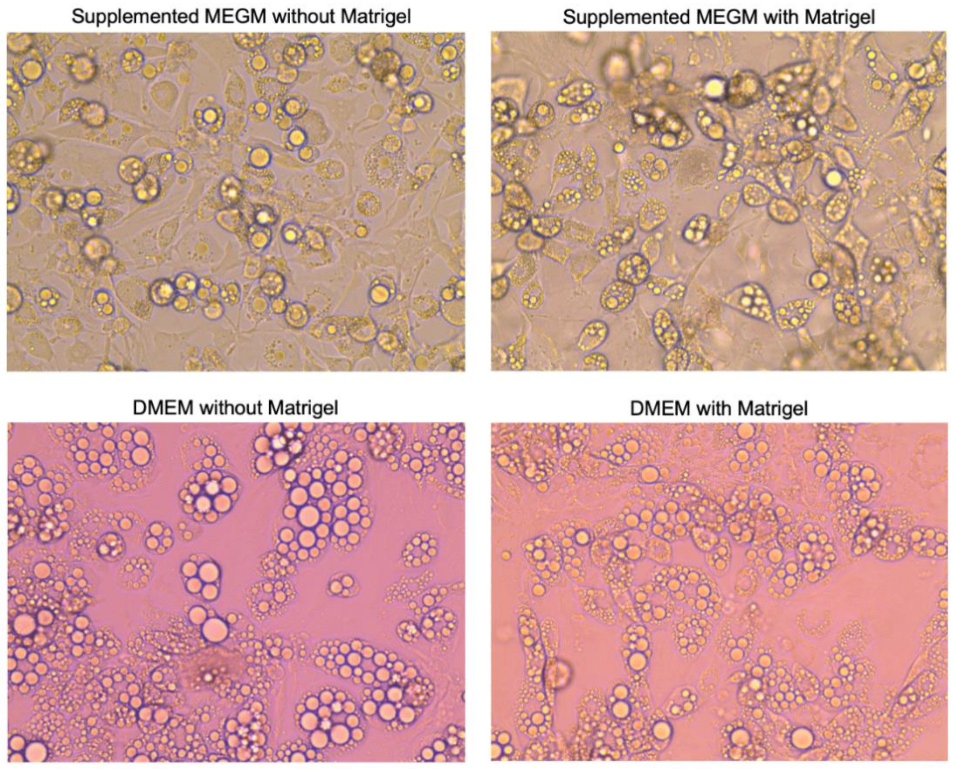

Figure S2. Representative images of mature adipocytes cultured in two media types, as well as in the presence and absence of Matrigel. Full differentiation of adipocytes can be visually verified, with ≥ 40% of cells containing lipid droplets. Magnification = 400×

Table S1. List of proteins differentially secreted by adipocytes in the presence of ECM (AE-CM) compared to adipocytes alone (A-CM) that are not reported to be in Matrigel (PSM counts > 2 in AE-CM, fold change > 2)

**Protein name**	**Symbol**	**A-CM (PSM counts)**	**AE-CM (PSM counts)**	**Fold Change**	**Function according to STRING**
Nucleoprotein TPR (Translocated Promoter	Tpr	18.5	56.5	3.1	Nucleoprotein TPR; component of the nuclear pore complex (NPC), a complex required for the trafficking across the nuclear envelope. Functions as a scaffolding element

Region, Nuclear Basket Protein)					in the nuclear phase of the NPC essential for normal nucleocytoplasmic transport of proteins and mRNAs, plays a role in the establishment of nuclear-peripheral chromatin compartmentalization in interphase, and in the mitotic spindle checkpoint signaling during mitosis. Involved in the quality control and retention of unspliced mRNAs in the nucleus; in association with NUP153, regulates the nuclear export of unspliced mRNA [...] (2363 aa)
Stress-70 protein, mitochondrial	Hspa9	10.5	27	2.6	Stress-70 protein, mitochondrial; chaperone protein which plays an important role in mitochondrial iron-sulfur cluster (ISC) biogenesis. Interacts with and stabilizes ISC cluster assembly proteins FXN, NFU1, NFS1 and ISCU. Regulates erythropoiesis via stabilization of ISC assembly. May play a role in the control of cell proliferation and cellular aging (by similarity); belongs to the heat shock protein 70 family (679 aa)
14-3-3 protein epsilon	Ywhae	nd	16.5		14-3-3 protein epsilon; adapter protein implicated in the regulation of a large spectrum of both general

					and specialized signaling pathways. Binds to a large number of partners, usually by recognition of a phosphoserine or phosphothreonine motif. Binding generally results in the modulation of the activity of the binding partner (by similarity). Positively regulates phosphorylated protein HSF1 nuclear export to the cytoplasm; belongs to the 14-3-3 family (255 aa)
UDP-glucose:glycoprotein glucosyltransferase 1	Uggt1	nd	16		UDP-glucose-glycoprotein glucosyltransferase 1; recognizes glycoproteins with minor folding defects. Reglucosylates single N-glycans near the misfolded part of the protein, thus providing quality control for protein folding in the endoplasmic reticulum. Reglucosylated proteins are recognized by calreticulin for recycling to the endoplasmic reticulum and refolding or degradation; UDP-glucose glycoprotein glucosyltransferases (1555 aa)
Polyadenylate-binding protein 1	Pabpc1	nd	15.5		Polyadenylate-binding protein 1; binds the poly(A) tail of mRNA, including that of its own transcript. May be involved in cytoplasmic regulatory processes of mRNA

					metabolism such as pre-mRNA splicing. Its function in translational initiation regulation can either be enhanced by or repressed by PAIP2. Can probably bind to cytoplasmic RNA sequences other than poly(A) in vivo. Involved in translationally coupled mRNA turnover. Implicated with other RNA-binding proteins in the cytoplasmic deadenylation/translational and decay interplay of the FOS mRNA mediated by the major coding-r [...] (636 aa)
Proliferating cell nuclear antigen	Pcna	nd	13		Proliferating cell nuclear antigen; auxiliary protein of DNA polymerase delta and is involved in the control of eukaryotic DNA replication by increasing the polymerase's processibility during elongation of the leading strand. Induces a robust stimulatory effect on the 3'-5' exonuclease and 3'-phosphodiesterase, but not apurinic-apyrimidinic (AP) endonuclease, APEX2 activities. Has to be loaded onto DNA in order to be able to stimulate APEX2. Plays a key role in DNA damage response (DDR) by being conveniently positioned at the replication fork to coordinate DNA

					replication with DNA re [...] (261 aa)
Endoplasmic reticulum resident protein 44	Erp44	nd	10	10.0	Endoplasmic reticulum resident protein 44; mediates thiol-dependent retention in the early secretory pathway, forming mixed disulfides with substrate proteins through its conserved CRFS motif. Inhibits the calcium channel activity of ITPR1. May have a role in the control of oxidative protein folding in the endoplasmic reticulum. Required to retain ERO1A and ERO1B in the endoplasmic reticulum (406 aa)
Glucosamine-6-phosphate isomerase 1	Gnpda1	3	8.5	2.8	Glucosamine-6-phosphate isomerase 1; seems to trigger calcium oscillations in mammalian eggs. These oscillations serve as the essential trigger for egg activation and early development of the embryo (by similarity); belongs to the glucosamine/galactosamine-6-phosphate isomerase family (289 aa)
Protein FAM49B	Fam49b	2.5	7	2.8	Protein FAM49B; family with sequence similarity 49 member B (324 aa)
KH domain-containing	Khdrbs1	nd	6.5		KH domain-containing, RNA-binding, signal transduction-associated protein 1; recruited and

, RNA-binding, signal transduction-associated protein 1 (band 2)					tyrosine phosphorylated by several receptor systems, for example the T-cell, leptin and insulin receptors. Once phosphorylated, functions as an adapter protein in signal transduction cascades by binding to SH2 and SH3 domain- containing proteins. Role in G2-M progression in the cell cycle. Represses CBP-dependent transcriptional activation apparently by competing with other nuclear factors for binding to CBP. Also acts as a putative regulator of mRNA stability and/or translation rates and mediate [...] (443 aa)
Isoform 2 of AP-2 complex subunit beta	Ap2b1	nd	6		AP-2 complex subunit beta; component of the adaptor protein complex 2 (AP-2). Adaptor protein complexes function in protein transport via transport vesicles in different membrane traffic pathways. Adaptor protein complexes are vesicle coat components and appear to be involved in cargo selection and vesicle formation. AP-2 is involved in clathrin-dependent endocytosis in which cargo proteins are incorporated into vesicles surrounded by clathrin (clathrin-coated vesicles, CCVs) which are

					destined for fusion with the early endosome. The clathrin lattice serves as a mechanical scaffold bu [...] (951 aa)
TFG protein (band 1)	Tfg	nd	5		Protein TFG; plays a role in the normal dynamic function of the endoplasmic reticulum (ER) and its associated microtubules (400 aa)
U2 snRNP-associated SURP motif-containing protein	U2surp	nd	4.5		U2 snRNP-associated SURP motif-containing protein; U2 snRNP associated SURP domain containing; belongs to the splicing factor SR family (1029 aa)
WD repeat-containing protein 5	Wdr5	nd	4.5		WD repeat-containing protein 5; contributes to histone modification. May position the N- terminus of histone H3 for efficient trimethylation at ‘Lys-4’. As part of the MLL1/MLL complex it is involved in methylation and dimethylation at ’Lys-4’ of histone H3. H3 ’Lys-4’ methylation represents a specific tag for epigenetic transcriptional activation. As part of the NSL complex it may be involved in acetylation of nucleosomal histone H4 on several lysine residues. May regulate osteoblasts differentiation;

					belongs to the WD repeat WDR5/wds family (334 aa)
Guanine nucleotide-binding protein G(s) subunit alpha isoforms XLas	Gnas	nd	4		Guanine nucleotide-binding protein G(s) subunit alpha isoforms XLas; guanine nucleotide-binding proteins (G proteins) function as transducers in numerous signaling pathways controlled by G protein-coupled receptors (GPCRs). Signaling involves the activation of adenylyl cyclases, resulting in increased levels of the signaling molecule cAMP. GNAS functions downstream of several GPCRs, including beta-adrenergic receptors. XLas isoforms interact with the same set of receptors as GNAS isoforms (by similarity) (1037 aa)
Prolyl 3-hydroxylase 3	P3h3	nd	4		Prolyl 3-hydroxylase 3; part of a complex composed of PLOD1, P3H3 and P3H4 that catalyzes hydroxylation of lysine residues in collagen alpha chains and is required for normal assembly and cross-linkling of collagen fibrils. Required for normal hydroxylation of lysine residues in type I collagen chains in skin, bone, tendon, aorta and cornea. Required for normal skin stability via its role in hydroxylation of lysine residues in

					collagen alpha chains and in collagen fibril assembly. Apparently not required for normal prolyl 3-hydroxylation on collagen chains, possibly because it function [...] (736 aa)
Isoform 2 of Active breakpoint cluster region-related protein	Abr	1.0	3.5	3.5	Active breakpoint cluster region-related protein; GTPase-activating protein for RAC and CDC42. Promotes the exchange of RAC or CDC42-bound GDP by GTP, thereby activating them; C2 domain containing (859 aa)
AP-1 complex subunit gamma	Ap1g1	nd	3.5		AP-1 complex subunit gamma-1; subunit of clathrin-associated adaptor protein complex 1 that plays a role in protein sorting in the late-Golgi/trans-Golgi network (TGN) and/or endosomes. The AP complexes mediate both the recruitment of clathrin to membranes and the recognition of sorting signals within the cytosolic tails of transmembrane cargo molecules (825 aa)
E3 ubiquitin-protein ligase RNF181	Rnf181	1.5	3	2.0	E3 ubiquitin-protein ligase RNF181; E3 ubiquitin-protein ligase which accepts ubiquitin from an E2 ubiquitin-conjugating enzyme in the form of a thioester and then directly transfers the

					ubiquitin to targeted substrates; belongs to the RNF181 family (153 aa)
Far upstream element-binding protein 1	Fubp1	nd	3		Far upstream element-binding protein 1; regulates MYC expression by binding to a single-stranded far-upstream element (FUSE) upstream of the MYC promoter. May act both as activator and repressor of transcription (644 aa)
KH domain-containing, RNA-binding, signal transduction-associated protein 1 (band 1)	Khdrbs1	nd	3		KH domain-containing, RNA-binding, signal transduction-associated protein 1; recruited and tyrosine phosphorylated by several receptor systems, for example the T-cell, leptin and insulin receptors. Once phosphorylated, functions as an adapter protein in signal transduction cascades by binding to SH2 and SH3 domain- containing proteins. Role in G2-M progression in the cell cycle. Represses CBP-dependent transcriptional activation apparently by competing with other nuclear factors for binding to CBP. Also acts as a putative regulator of mRNA stability and/or translation rates and mediate [...] (443 aa)
Mitogen-activated	Mapk1	nd	2.5		Mitogen-activated protein kinase 1; serine/threonine kinase which acts

protein kinase 1					as an essential component of the MAP kinase signal transduction pathway. MAPK1/ERK2 and MAPK3/ERK1 are the 2 MAPKs which play an important role in the MAPK/ERK cascade. They participate also in a signaling cascade initiated by activated KIT and KITLG/SCF. Depending on the cellular context, the MAPK/ERK cascade mediates diverse biological functions such as cell growth, adhesion, survival and differentiation through the regulation of transcription, translation, cytoskeletal rearrangements. The MAPK/ERK cascade plays a [...] (360 aa)
BTB/POZ domain-containing protein KCTD12	Kctd12	1.0	2.0	2.0	BTB/POZ domain-containing protein KCTD12; auxiliary subunit of GABA-B receptors that determine the pharmacology and kinetics of the receptor response. Increases agonist potency and markedly alter the G-protein signaling of the receptors by accelerating onset and promoting desensitization (by similarity) (325 aa)
Isoform 2 of	Sgce	1	2	2.0	Epsilon-sarcoglycan; component of the sarcoglycan complex, a

Epsilon-sarcoglycan					subcomplex of the dystrophin-glycoprotein complex which forms a link between the F-actin cytoskeleton and the extracellular matrix (462 aa)
Toll-interacting protein	Tollip	1	2	2.0	Toll-interacting protein; component of the signaling pathway of IL-1 and Toll-like receptors. Inhibits cell activation by microbial products. Recruits IRAK1 to the IL-1 receptor complex. Inhibits IRAK1 phosphorylation and kinase activity. Connects the ubiquitin pathway to autophagy by functioning as a ubiquitin- ATG8 family adapter and thus mediating autophagic clearance of ubiquitin conjugates. The TOLLIP-dependent selective autophagy pathway plays an important role in clearance of cytotoxic polyQ proteins aggregates; C2 domain containing (274 aa)
V-type proton ATPase subunit E 1	Atp6v1e1	nd	2		V-type proton ATPase subunit E 1; subunit of the peripheral V1 complex of vacuolar ATPase essential for assembly or catalytic function. V-ATPase is responsible for acidifying a variety of intracellular compartments in

					eukaryotic cells; V-type ATPases (226 aa)
Protein-lysine 6-oxidase	Lox	nd	2		Protein-lysine 6-oxidase; responsible for the post-translational oxidative deamination of peptidyl lysine residues in precursors to fibrous collagen and elastin. Regulator of Ras expression. May play a role in tumor suppression. Plays a role in the aortic wall architecture (by similarity); belongs to the lysyl oxidase family (417 aa)
Mitogen-activated protein kinase 14	Mapk14	nd	2		Mitogen-activated protein kinase 14; serine/threonine kinase which acts as an essential component of the MAP kinase signal transduction pathway. MAPK14 is one of the four p38 MAPKs which play an important role in the cascades of cellular responses evoked by extracellular stimuli such as proinflammatory cytokines or physical stress leading to direct activation of transcription factors. Accordingly, p38 MAPKs phosphorylate a broad range of proteins and it has been estimated that they may have approximately 200 to 300 substrates each. Some of

					the targets are downstream kinases which are a [...] (360 aa)
Prolyl 3-hydroxylase 2	P3h2	nd	2		Prolyl 3-hydroxylase 2; prolyl 3-hydroxylase that catalyzes the post-translational formation of 3-hydroxyproline on collagens. Contributes to proline 3-hydroxylation of collagen COL4A1 and COL1A1 in tendons, the eye sclera and in the eye lens capsule (By similarity). Has high activity with the type IV collagen COL4A1, and lower activity with COL1A1. Catalyzes hydroxylation of the first Pro in Gly-Pro-Hyp sequences where Hyp is 4-hydroxyproline. Has no activity on substrates that lack 4- hydroxyproline in the third position; belongs to the leprecan family (708 aa)
Formylglycine-generating enzyme	Sumf1	nd	2		Sulfatase-modifying factor 1; Using molecular oxygen and an unidentified reducing agent, oxidizes a cysteine residue in the substrate sulfatase to an active site 3-oxoalanine residue, which is also called C(alpha)-formylglycine. Known substrates include GALNS, ARSA, STS and ARSE; belongs to the sulfatase-modifying factor family (374 aa)

Transcriptional enhancer factor TEF-1	Tead1	nd	2		Transcriptional enhancer factor (TEF)-1; transcription factor which plays a key role in the Hippo signaling pathway, a pathway involved in organ size control and tumor suppression by restricting proliferation and promoting apoptosis. The core of this pathway is composed of a kinase cascade wherein MST1/MST2, in complex with its regulatory protein SAV1, phosphorylates and activates LATS1/2 in complex with its regulatory protein MOB1, which in turn phosphorylates and inactivates YAP1 oncoprotein and WWTR1/TAZ. Acts by mediating gene expression of YAP1 and WWTR1/TAZ, thereby regulating cell [...] (426 aa)
TFG protein (band 2)	Tfg	nd	2		Protein TFG; plays a role in the normal dynamic function of the endoplasmic reticulum (ER) and its associated microtubules (400 aa)

PSM: peptide-spectrum matches; [...]: indicates that the description continues
